# Surface Modification of Screen-Printed Carbon Electrode through Oxygen Plasma to Enhance Biosensor Sensitivity

**DOI:** 10.3390/bios14040165

**Published:** 2024-03-29

**Authors:** Shuto Osaki, Masato Saito, Hidenori Nagai, Eiichi Tamiya

**Affiliations:** 1 AIST-Osaka University Advanced Photonics and Biosensing Open Innovation Laboratory, National Institute of Advanced Industrial Science and Technology, Photonics Center, Osaka University, 2-1 Yamadaoka, Suita 565-0871, Osaka, Japanhide.nagai@aist.go.jp (H.N.); 2 Graduate School of Engineering, Osaka University, 2-1 Yamadaoka, Suita 565-0871, Osaka, Japan; 3 SANKEN-The Institute of Scientific and Industrial Research, Osaka University, 8-1 Mihogaoka, Ibaraki 567-0047, Osaka, Japan

**Keywords:** electrochemical immunosensor, gold nanoparticles, oxygen plasma, point-of-care, biosensors

## Abstract

The screen-printed carbon electrode (SPCE) is a useful technology that has been widely used in the practical application of biosensors oriented to point-of-care testing (POCT) due to its characteristics of cost-effectiveness, disposability, miniaturization, wide potential window, and simple electrode design. Compared with gold or platinum electrodes, surface modification is difficult because the carbon surface is chemically or physically stable. Oxygen plasma (O_2_) can easily produce carboxyl groups on the carbon surface, which act as scaffolds for covalent bonds. However, the effect of O_2_-plasma treatment on electrode performance remains to be investigated from an electrochemical perspective, and sensor performance can be improved by clarifying the surface conditions of plasma-treated biosensors. In this research, we compared antibody modification by plasma treatment and physical adsorption, using our novel immunosensor based on gold nanoparticles (AuNPs). Consequently, the O_2_-plasma treatment produced carboxyl groups on the electrode surface that changed the electrochemical properties owing to electrostatic interactions. In this study, we compared the following four cases of SPCE modification: O_2_-plasma-treated electrode/covalent-bonded antibody (a); O_2_-plasma-treated electrode/physical adsorbed antibody (b); bare electrode/covalent-bonded antibody (c); and bare electrode/physical absorbed antibody (d). The limits of detection (LOD) were 0.50 ng/mL (a), 9.7 ng/mL (b), 0.54 ng/mL (c), and 1.2 ng/mL (d). The slopes of the linear response range were 0.039, 0.029, 0.014, and 0.022. The LOD of (a) was 2.4 times higher than the conventional condition (d), The slope of (a) showed higher sensitivity than other cases (b~d). This is because the plasma treatment generated many carboxyl groups and increased the number of antibody adsorption sites. In summary, the O_2_-plasma treatment was found to modify the electrode surface conditions and improve the amount of antibody modifications. In the future, O_2_-plasma treatment could be used as a simple method for modifying various molecular recognition elements on printed carbon electrodes.

## 1. Introduction

The screen-printed carbon electrode (SPCE) is a useful technology that has been widely used in the practical application of biosensors oriented toward point-of-care testing (POCT) [1,2,3,4,5,6,7]. Many researchers have reported on electrochemical biosensors that use SPCE to detect hormones [8,9,10], ions [11,12], metals [13], nucleic acid [14,15,16,17,18,19], and proteins [20,21,22,23]. Although the sensitivity of the electrochemical biosensor is controlled by surface conditions, such as the diffusion coefficient and electron transfer rate [24], these are altered by the modification of molecular recognition elements and blocking materials on the electrode surface. We previously reported on biosensors that use redox reactions of gold nanoparticles and found that antibodies and blocking materials modified on the electrode reduced the diffusion coefficient and electron transfer rate [25]. As it is necessary to measure very small amounts of biomarkers to achieve POCT, electrochemical biosensors with higher sensitivity and selectivity are required. Therefore, a more efficient method for modifying molecular recognition elements, such as antibodies, without degrading their electrochemical properties is required. However, compared to gold or platinum electrodes, the surface modification of SPCEs is difficult because the carbon surface is chemically or physically stable [26]. For gold or platinum electrodes, a self-assembled monolayer (SAM) based on alkanethiol is mainly used for antibody modification [27,28,29,30,31,32]. Aptamers modified with a thiol group are also used, based on the same principle [33]. In addition, drop-casted biopolymers and nanomaterials do not depend on the electrode material [16,34,35,36,37,38,39,40,41].

For the carbon electrode, the direct production of a carboxyl group, which acts as a scaffold for the covalent bonds on the carbon surface using electrochemical activation, has been reported [42,43,44]. For example, the SPCE surface was activated to produce a carboxyl group by applying a potential of 0.9 V for 60 s in a 0.5 M acetate buffer (ABS, pH 4.80) [45] or 1.0 V for 50 s in a 0.10 M sulfuric acid solution [46]. Oxygen plasma (O_2_-plasma) treatment is an efficient technique for producing carboxyl groups on the surface [47,48,49] and is used for surface cleaning and regeneration [50,51].

However, regarding SPCE, there have been no reports of antibody modification by generating carboxyl groups using O_2_-plasma treatment, and only improvements in electrochemical performance have been discussed. For example, O_2_ plasma is used to remove the binder from carbon inks and control the surface roughness of SPCE [52,53,54,55,56,57].

In this study, we compared antibody modification by O_2_-plasma treatment and physical adsorption using our novel immunosensor, which is a gold-linked electrochemical immunoassay (GLEIA). This biosensor is based on a sandwich-type immunoassay applied directly on the electrode and detects the antigen concentration through the redox current of gold nanoparticles (AuNPs) modified with a secondary antibody [5,25,58,59,60,61,62]. Specifically, the AuNPs on the electrode are oxidized at a high potential to produce gold ions, and the concentration of nanoparticles is quantified by measuring the reduction current of gold ions. The chemical reaction is Au + Cl_4_ ⇄ AuCl_4_ + 3e (E_0_ = 0.803 V, vs. Ag/AgCl sat.). We previously reported that physically adsorbed antibodies decrease the electrochemical reaction rate because they act as a resistance on the electrode [25]. Thus, O_2_-plasma and covalent bonding reagents can be used as an alternative antibody-modification method to physical adsorption. We investigated the O_2_-plasma-treated surface by cyclic voltammetry (CV), scanning electron microscopy (SEM), X-ray photoelectron spectroscopy (XPS), and contact angle analysis. Consequently, the generation of carboxyl groups on the electrode surface, changes in the surface charge, increased capacitance, and hydrophilicity were observed. These changes can be explained by the generation of carboxyl groups on the electrode surface, indicating that O_2_-plasma treatment is a simple and effective surface modification method. We also observed that the O_2_-plasma-treated electrode showed higher sensitivity than the electrode without O_2_-plasma for immunoglobulin A (IgA) because the covalently bonded antibody inhibited nonspecific adsorption with the abovementioned changes on the surface.

## 2. Materials and Methods

### 2.1. Reagents

All reagents used were of guaranteed grade and used without further purification. All inorganic salts, 1-Ethyl-3-(3dimethylaminopropyl) carbodiimide ·HCl (EDC), N-hydroxysuccinimide (NHS), polyethylene glycol (Mw is 20,000), trehalose dihydrate, and pH 7.5 D-PBS (-) were purchased from Fujifilm Wako Pure Chemicals (Osaka, Japan). AuNPs with diameters of 60 nm were purchased from BBI Solutions (Cardiff, UK). Anti-IgA (α), Human, Goat-Poly, A80-102A, and Purified Human IgA, P80-102 were purchased from Bethyl Laboratory (Montgomery, AL, USA). Bovine serum albumin (BSA) was purchased from Jackson Immunoresearch (West Grove, PA, USA). All water used in this study was Milli-Q water (18.3 MΩ cm).

### 2.2. Instruments

A miniSTAT400 potentiostat was purchased from BioDevice Technology (Ishikawa, Japan). A disposable screen-printed carbon electrode (DEP-EP-PP) with an integrated working electrode (surface area: 2.64 mm^2^), counter electrode, and Ag/AgCl reference electrode, with a total length of 11 mm, was also purchased from BioDevice Technology. All electrochemical measurements were performed by dropping 20 µL of the sample onto the printed electrode, unless otherwise stated. A UV-visible spectrometer (DS-11+) was purchased from Denovix (Wilmington, DE, USA). A micro-high-speed cooling centrifuge (kitman-24) was purchased from Tomy Seiko (Tokyo, Japan). An O_2_-plasma cleaner (PDC210) was purchased from Yamato Scientific (Tokyo, Japan). Scanning electron microscopy (SEM) was performed using an S-4800 (Hitachi High-Tech, Tokyo, Japan). X-ray photoelectron spectroscopy (XPS) analysis was performed by Toray Research Center (Tokyo, Japan) using a Quantera SXM (Ulvac-PHI). Contact angle analysis was performed using a DMo-602 instrument obtained from Kyowa Interface Science (Saitama, Japan). Electrochemical Impedance Spectroscopy was performed by Autolab PGSTAT 14N (Metrohm Autolab Utrecht, Kanaalweg 29G, Utrecht, The Netherlands).

### 2.3. Electrochemical Analysis of O_2_-Plasma-Treated Electrodes

The O_2_-plasma treatment was performed in a 13.56 MHz ratio frequency (RF) plasma reactor. The SPCE, with a cyclo-olefin polymer (COP) film covering the connector part and reference electrode, was placed in the reactor. After the reactor was first evacuated to a base pressure of less than 10^−3^ Pa, 200 cc O_2_ gas was introduced. The O_2_-plasma treatment of the SPCE was performed at 75 W plasma power for a period of 5 s. The electrode was evaluated using cyclic voltammetry (CV) with the standard electrochemical mediators: potassium ferricyanide and hexaammineruthenium(III) chloride, each containing 100 mM of Na_2_SO_4_ as the electrolyte,. For the ferricyanide, the sweep rates were 10 to 250 mV/s, and the sweep range was −400 to 600 mV. For the ruthenium, the sweep rates were 10 to 200 mV/s, and the sweep range was 700 to −700 mV. Electrochemical impedance spectroscopy (EIS) was also used to evaluate the kinetic parameters within the frequency range of 100 kHz to 0.1 Hz applied potential, superimposed on a DC potential of 0.1 V, with an AC of 10 mV peak-to-peak amplitude under 5 mM ferricyanide and 100 mM Na_2_SO_4_.

### 2.4. Surface Analysis of O_2_-Plasma-Treated Electrodes

The electrode surface was analyzed by SEM, XPS, and contact angle measurement. SEM was used to observe the electrode surface after the antigen-antibody reaction to evaluate the AuNPs present on the surface. For this purpose, X-rays (monochromatic Al K∝ ray) of 200 µm diameter were irradiated on the electrodes, which were stored in light-shielded vacuum gauges prior to the XPS analysis. The contact angle measurement was conducted using water drops (2 µL). The drops were placed on the working electrode, the needle was pulsed back, and the drop shape was immediately captured using the camera. The obtained images from the electrodes were analyzed using FAMAS software (ver. 7.2.0 from Kyowa Interface Science Co., Ltd.) to determine the circle fitting.

### 2.5. Antibody Modification

**Scheme 1:**
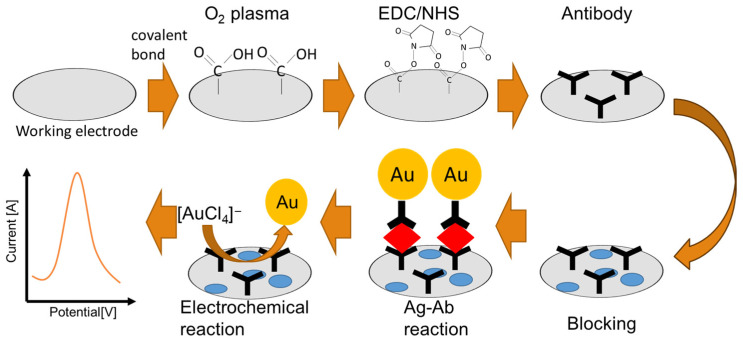
Schematic illustration of the biosensor fabrication and sensing flow.

The electrode surface was modified via physical adsorption and covalent bonding to compare the response of the sensor to the antibody modification process. For the physical adsorption, 2 µL of 50 µg/mL antibody in PBS was dropped onto the working electrode and incubated at 4 °C for 1 h to adsorb the antibody. Next, 20 µL of 1% BSA in PBS solution was dropped onto the entire electrode and incubated at room temperature (RM) for 1 h. For covalent bonding, 50:50 mM of EDC/NHS solution was dropped onto the working electrode and incubated for 30 min. Next, 2 µL of 50 ug/mL antibody in PBS was placed on the electrodes and incubated for 30 min. Finally, 20 µL of 1% BSA in PBS solution was dropped onto the entire electrode and incubated at RM for 30 min (see Scheme 1). The prepared electrode was stored at 4 °C until use.

### 2.6. Preparing Secondary Antibody-Modified Gold Nanoparticles

Secondary antibody-modified AuNPs were prepared using a reported method [5]. Then, the 0.9 mL of AuNP solution was mixed with the 0.1 mL of phosphate buffer (Na_2_HPO_4_/NaH_2_PO_4_, 50 mM, pH 7.5). 50 µg/mL of Anti-IgA was added to the Au nanoparticle solution to dissolve the 5 mM of phosphate buffer (pH 7.5) and kept for 10 min at RM. Hereinafter, this is referred to as the Au conjugate. Then, 0.1 mL of 10% BSA in PBS buffer and 0.05 mL of 1% PEG in PBS buffer were added to the Au conjugate. The Au Anti-IgA conjugate was collected by centrifugal operation (4000× *g* for 20 min at 4 °C). After centrifugation, the Au Anti-IgA conjugate was suspended in 1 mL of preservation solution (1% BSA, 0.05% PEG 20000, 0.1% NaN_3_, and 150 mM NaCl in 20 mM Tris-HCl buffer, pH 8.2) and collected again in the same manner. For the stock solution, the Au Anti-IgA conjugate was suspended in the preservation solution, and the optical density was adjusted to OD_520_ = 6. The Au Anti-IgA conjugate was diluted 3 times with 50 *w/v* of trehalose (OD_520_ = 2) and by dripping 5 µL of this solution in the 96 wells. Then, the 96-well plate was dried in a vacuum condition for 5 min. Dried wells were stored at 4 °C until use.

### 2.7. Immunosensor Fabrication Using O_2_-Plasma-Treated SPCE

A sandwich type antigen–antibody (Ab–Ag) reaction occurred directly on the working electrode (Scheme 1). IgA antigen solutions (100–0 ng/mL) were prepared by dilution in PBS. A total of 10 µL of the IgA solution was dropped in the prepared 96-well plate and mixed for 10 s. After 1 min, 2.0 µL of the solution was placed on the working electrode to incubate for 1 h at RM in the closed box with damped cotton (to maintain humidity to prevent the sample from drying). After rinsing with PBS and eliminating the PBS solution with N_2_ air, the direct redox reaction was performed in 2 M KCl solution (20 µL) covering the entire electrode at RM. The pre-oxidation and differential pulse voltammetry (DPV) parameters were as follows: the beginning and end potentials were 800 and 200 mV, the step potential was 4 mV, pulse amplitude (pulse potential) was 100 mV, pulse period was 200 ms, pulse width was 50 ms, and sampling width was set to 16 ms. All conditions were optimized in our previous work [25].

## 3. Results and Discussion

### 3.1. Comparing the Surface Change by Electrochemical Kinetics Parameters

**Figure 1:**
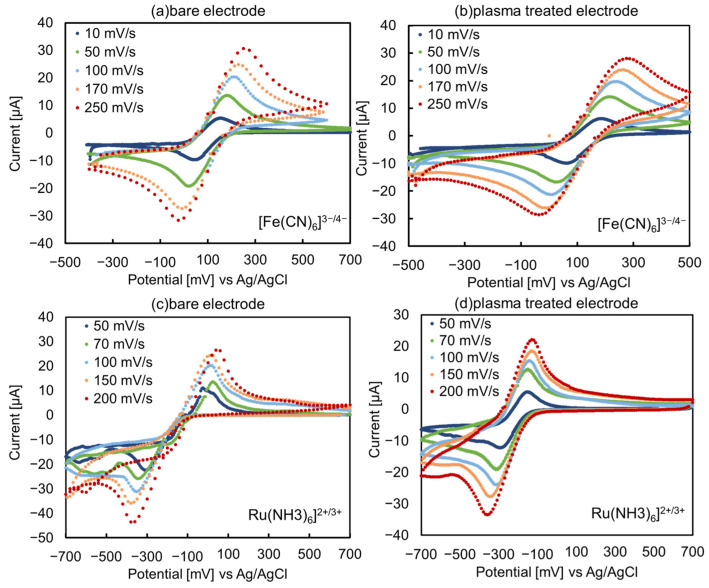
Cyclic voltammograms of 5 mM ferricyanide and 5 mM ruthenium complex on the bare (**a**,**c**); and plasma-treated electrodes (**b**,**d**).

**Figure 2:**
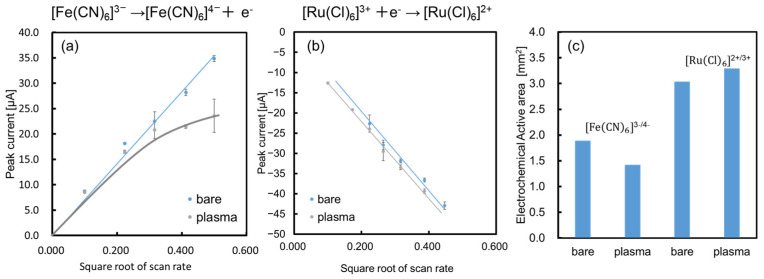
Relationship between peak current intensity and square root of scan rate for ferricyanide (**a**); and ruthenium complex (**b**). Electrochemical active areas of ferricyanide and ruthenium complex on bare electrode and O_2_-plasma-treated electrode (**c**).

Figure 1a–d shows the cyclic voltammograms of 5 mM ferricyanide and ruthenium complex on the bare and O_2_-plasma-treated electrodes. Among these voltammograms, the electrochemical reaction is a reversible process because of the peak separations at the 50 mV/s scan rate of 152, 190, 286, and 178 mV. Figure 2a,b shows the relationship between the peak current intensity and the square root of the scan rate. The diffusion coefficient for the reversible process was calculated using the following equation:

(1) $$I_{p,cv}=-\left(2.69\times 10^{5}\right)n^{\frac{3}{2}}AC_{bulk}D^{\frac{1}{2}}v^{\frac{1}{2}}$$

where n is the number of electrons in the reaction, A is the electrode area, *C_bulk_* is the concentration of the electrochemical mediators, and *v* is the scan rate.

The electroactive area of the two types of electrochemical mediators (ferricyanide and ruthenium complex) at both electrodes (bare and plasma) were calculated using Equation (1) and the diffusion coefficients were determined with results from a previous study. Both of the diffusion coefficients are $7.75\times 10^{-6}\;{cm}^{2}/s$ for ferricyanide, and $5.77\times 10^{-6}\;{cm}^{2}/s$ for ruthenium complex obtained from [63]. The effective electroactive areas ($A_{bare,\;Fe}$, $A_{plasma,\;Fe}$ and $A_{bare,Ru\;}$ and $A_{plasma,\;Ru}$) were 1.89 mm^2^, 1.42 mm^2^, 3.04 mm^2^ and 3.29 mm^2^, as summarized in Figure 2c.

From the results, it was observed that the electroactivity of the negatively charged ferricyanides decreased and that the positively charged ruthenium complexes slightly increased at the O_2_-plasma-treated electrode. Electrostatic repulsion for the ferricyanide may occur due to the negative charge of the carboxyl groups generated by the plasma treatment. For the positively charged ruthenium complex, instead of electrostatic repulsion not occurring, diffusion to the electrodes could have been promoted. Electrostatic repulsion occurs between dendrimers and electrochemical mediators on the electrode surface [64], indicating a close relationship between the surface charge and electrochemical activity.

**Figure 3:**
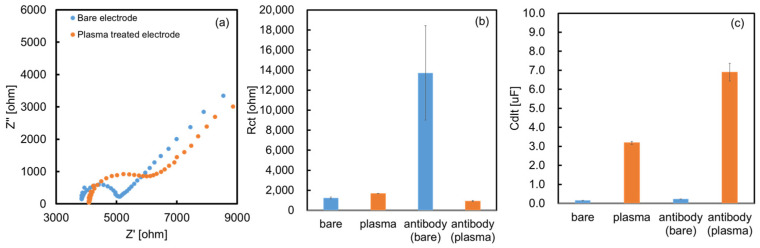
Cole-Cole plot of bare electrode and plasma-treated electrode in the 5 mM of ferricyanide (**a**); Rct value (**b**); and Cdl (**c**).

Next, charge transfer resistance (Rct) and capacitance (Cdl) were evaluated using EIS. Figure 3a shows the Cole-Cole plots of both electrodes in 5 mM of ferricyanide. The bare electrode showed a clear semicircle, while the O_2_-plasma-treated electrode showed partial disappearance of the semicircle. Based on these results, Cdl and Rct were obtained by fitting using the equivalent circuit. The plasma treatment did not change Rct, but increased Cdl (Figure 3b,c). This result supports the aforementioned surface charge change.

**Figure 4:**
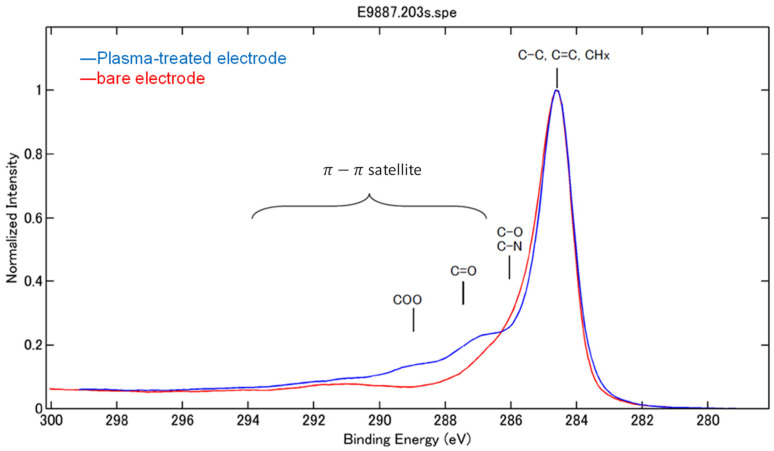
XPS spectra of plasma-treated electrode (blue line) and bare electrode (red line).

Therefore, the O_2_-plasma treatment changes the surface charge of the electrode and alters its electrochemical properties. Surface analysis using XPS suggested that this change in surface charge was due to the formation of carboxyl groups (Figure 4). Our results did not show any improvement in the electrode performance owing to the removal of impurities or binders in the SPCE. On the contrary, electrostatic interactions with reactive species and an improvement in hydrophilicity were observed due to the generation of the carboxyl groups. These results depend on the electrode materials and plasma irradiation conditions, indicating that individual optimization is required.

We also performed EIS measurements on the electrode after antibody modification. The results are shown in Figure 3b,c, where Rct increased when the antibody was modified by physical adsorption, and Cdl increased when it was modified by covalent bonding. These contrasting results are interesting and may indicate differences in the orientation and adsorption conditions of the antibody on each electrode. The antibody simply functions as an insulator because it is physically adsorbed on the bare electrodes and increases Rct. However, a gap occurs between the antibody and electrode because the antibody is modified by covalent bonding at the O_2_-plasma-treated electrode. Consequently, electrode resistance did not occur; instead, the electric double layer capacitance was altered by the electric charge of the antibody.

### 3.2. Sensor Characteristics

**Figure 5:**
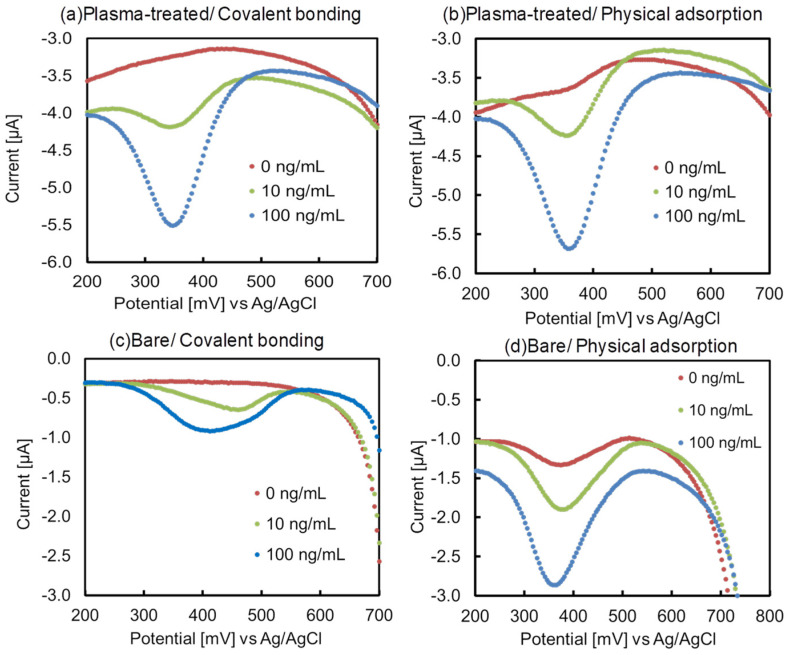
Differential pulse voltammograms of the pre-oxidized AuNPs immobilized on secondary antibody after sandwich immunoreaction in standard IgA solution (100, 10, 0 ng/mL) for plasma-treated/covalent bonding (**a**); plasma-treated electrode/physical adsorption (**b**); bare electrode/covalent bonding (**c**); and bare electrode/physical adsorption (**d**).

Figure 5 shows differential pulse voltammograms of the biosensor using O_2_-plasma-treated/covalent bonding (a), O_2_-plasma-treated electrode/physical adsorption (b), bare electrode/covalent bonding (c), and bare electrode/physical adsorption (d). Blank signals (0 ng/mL of IgA) were suppressed when covalent bonding was used (Figure 5a,c). The peak currents to 100 ng/mL of IgA were almost constant, except in Figure 5c.

The voltammograms also showed an increase in the background current at the O_2_-plasma-treated electrode. This may have been due to an increase in the charging current. In general, the charge current ($i_{charge}$) is given by Equation (2).

(2) $$i_{charge}=\frac{E}{Rs}\;e^{-\frac{t}{RsC_{dl}}}$$

where *E* is applied potential, *Rs* is solution resistance, and *t* is potential applied time.

In other words, the increase in background current is associated with an increase in Cdl. In addition, the Faraday current could be detected even when the charge current was increased. The results correlated with the relationship between the surface conditions and sensor sensitivity.

**Figure 6:**
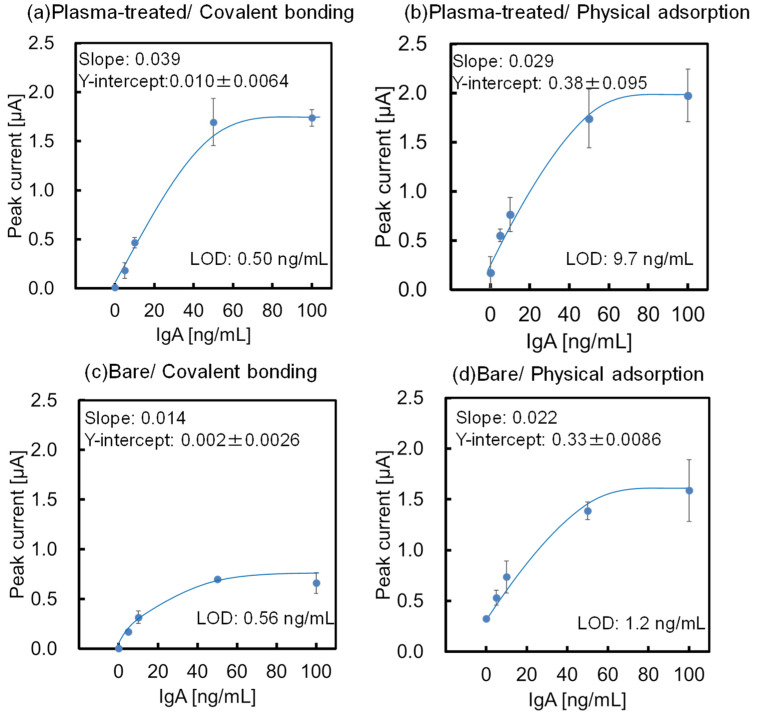
Calibration curves of IgA determination with the reduction peak intensity from pre-oxidized AuNP on plasma-treated electrode/covalent bonding (**a**); plasma-treated electrode/physical adsorption (**b**); bare electrode/covalent bonding (**c**); and bare electrode/physical adsorption (**d**).

The calibration curves obtained from each voltammogram are shown in Figure 6a–d. The process of calculating LOD was examined more precisely because of its importance. LOD is generally defined as three times the variation (3σ) of the blank measurement which is divided by the slope of the linear response range. In the present case, LOD was calculated assuming a linear response at 0~50 ng/mL. In addition, a weighted slope was used to account for variations at each concentration. As a result, the weighted slopes are 0.039 (a), 0.029 (b), 0.014 (c) and 0.022 (d). Y intercepts (blank signal) were 0.010 ± 0.0064 (a), 0.38 ± 0.095 (b), 0.002 ± 0.0026 (c), 0.33 ± 0.0086 (d). The calculated LODs are also 0.50 ng/mL (a), 9.7 ng/mL (b), 0.56 ng/mL (c) and 1.2 ng/mL (d), as shown in Figure 6.

Based on these results, we succeeded in increasing the sensitivity and LOD using an O_2_-plasma-treated electrode and covalent-bonded antibody.

This is due to the improvement in the surface conditions of the electrode and the suppression of non-specific adsorption by covalent-bonded antibody. Table S1 lists the signal values, averages, and standard deviations of each calibration curve typically picked up in Figure 6a,d that were used in the LOD calculations. This also suggested that physical adsorption is possible at the O_2_-plasma-treated electrodes (Figure 5b and Figure 6b). It was inferred that antibody adsorption is based on unstable electrostatic interaction rather than hydrophobic interaction, which induced nonspecific adsorption as in the bare electrode. In this case, the variation of the blank signal is large.

Figure 6c shows a very effective LOD (0.56 ng/mL) but a very poor slope of 0.014. This is presumably due to the low amount of carboxyl groups present on the surface, which prevented the reaction of EDC/NHS, resulting in insufficient modification of the antibody. We have investigated the selectivity using the same antibodies and antigens as in the present study. We also investigated real saliva samples [62] and saliva reproduced samples [5] using a biosensor based on the same principle. The biosensor based on the AuNP showed sufficient selectivity. Therefore, the blank signals obtained in this study were attributed to the modification of the surface state by plasma treatment and EDC/NHS.

**Figure 7:**
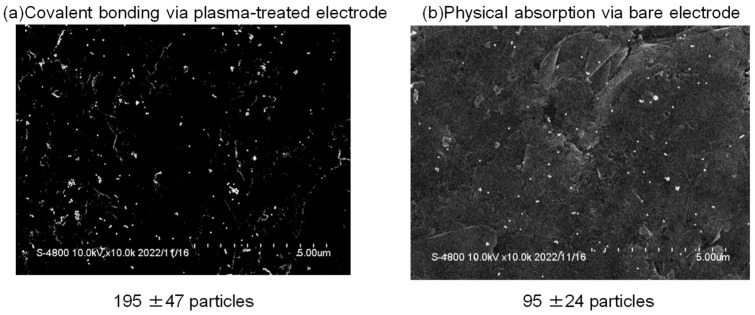
Scanning electron microscopy images observed from after antigen–antibody reaction on O_2_-plasma-treated electrode and covalent bonding (**a**); and bare electrode and physical adsorption (**b**).

We observed the electrode surface immediately after reaction with 100 ng/mL of IgA using SEM and found the presence of AuNP on the electrode (Figure 7). We have performed elemental analysis of these particles using SEM-EDX in a previous study and have shown that they are Au [25]. The number of AuNPs were counted: 195 ± 47 for the O_2_-plasma-treated electrode and 95 ± 24 for the bare electrode. This suggests that covalent bonding and O_2_-plasma treatment can improve the amount and orientation of the antibodies. However, the linear response range is always constant, as can be seen in Figure 6, suggesting that the dissociation constant is also the same without the surface condition. Typically, comparing Figure 6a,d, there is no significant change in the current values obtained at 100 ng/mL of IgA. This may be due to the reduced reactivity with the negatively charged molecules, as shown in Figure 2, and therefore also with the negatively charged gold complexes ([AuCl_4_]^−^). This result correlates with Figure S2, which investigated the sensitivity to particle number by AuNPs directly on the plasma treatment electrode. The LOD of 190,000 particles on the O_2_-plasma-treated electrode is well below the 6000 on the bare electrode that we previously reported [25].

In general, the SEM showed the amount and orientation of antibody modification, and the EIS showed the electrochemical activity of the electrode, revealing the effects of O_2_-plasma and covalent bonding on the antibody and electrode.

## 4. Conclusions

We investigated the modification of SPCE using O_2_-plasma to develop highly sensitive electrochemical biosensors. XPS analysis and contact angle measurements of the O_2_-plasma-treated electrode confirmed the generation of carboxyl groups and improved hydrophilicity. We also investigated the electrode surface using electrochemical methods such as CV and EIS. The results of both methods support the XPS and contact angle measurements, and the electrode surface was successfully modified. Therefore, we modified antibodies with common covalent bonding reagents, such as EDC/NHS, and four cases were compared: with and without O_2_-plasma, and with and without covalent bonding.

The improvement rate with the O2-plasma and covalent bonding was 2.4 times higher with regard to the LOD and 1.8 times higher with regard to the slope compared to the conventional method with the bare electrode and physical adsorption. The SEM images also suggested that antibody modification using covalent bonding on the plasma-treated electrode improved the amount of antibody modification. However, the electrochemical reactivity to the AuNPs was also reduced, limiting the improvement in sensitivity. As a result, we found a close relationship between the substituents, charge, and hydrophilicity of the electrode surface and electrochemical activity, which affects sensor sensitivity. Our findings relate to the relationship between the electrode surface conditions and sensor sensitivity and suggested that we might identify important factors in a discussion of antibody modification methods. There are a few simple and effective methods for surface modification of SPCE, including electrochemical activation, drop casting [65], and plasma treatment. By comparison, plasma treatment has the advantage of being able to treat many electrodes in a short time (5 s) and to generate carboxyl groups. It can also remove binders and dust from the electrode surface.

In the future, we plan to use the biosensors with improved sensitivity to measure previously undetectable biomarkers and develop the novel biosensors using the unique surface conditions generated by O_2_-plasma.

## Data Availability

The data that support the findings of this study are available from the corresponding authors upon reasonable request.

## Supplementary Materials

The following supporting information can be downloaded at: https://www.mdpi.com/article/10.3390/bios14040165/s1, Figure S1: Contact angle measurement of O_2_-plasma-treated electrode. Table S1: Signal values used in the calibration curves. Figure S2: Differential pulse voltammograms (a) and Calibration curve (b) for AuNP modified on SPCE.
