# Supplementary material for: Surface Modification of Screen-Printed Carbon Electrode through Oxygen Plasma to Enhance Biosensor Sensitivity
Source: Biosensors (Basel). 2024 Mar 29;14(4):165. doi: 10.3390/bios14040165 (PMC11048330; doi:10.3390/bios14040165)
Supplement: Supplementary file 1 [file biosensors-14-00165-s001.zip › biosensors-2872396-supplementary.pdf]

Supplementary Materials

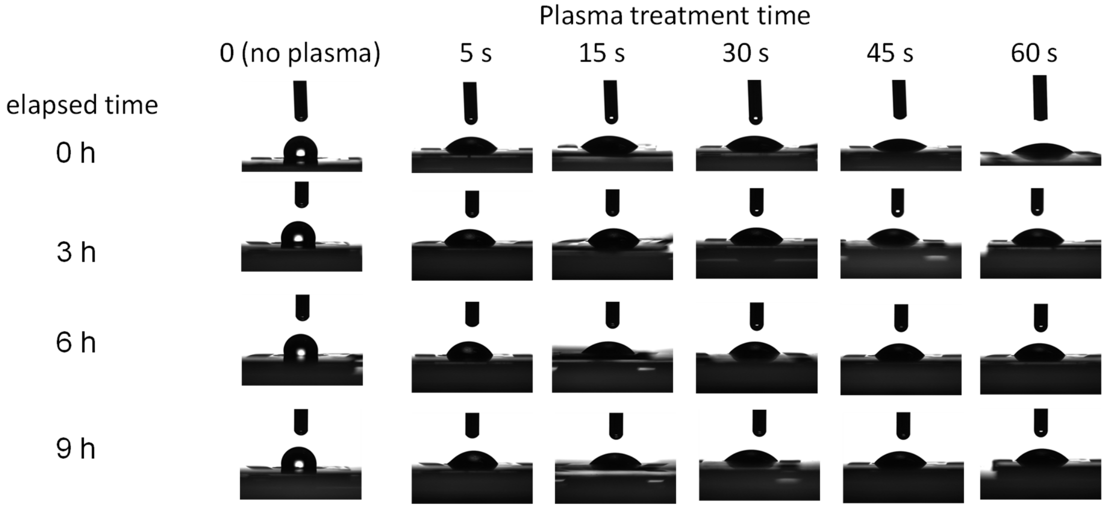

Figure S1: Contact angle measurement of plasma treated electrode.

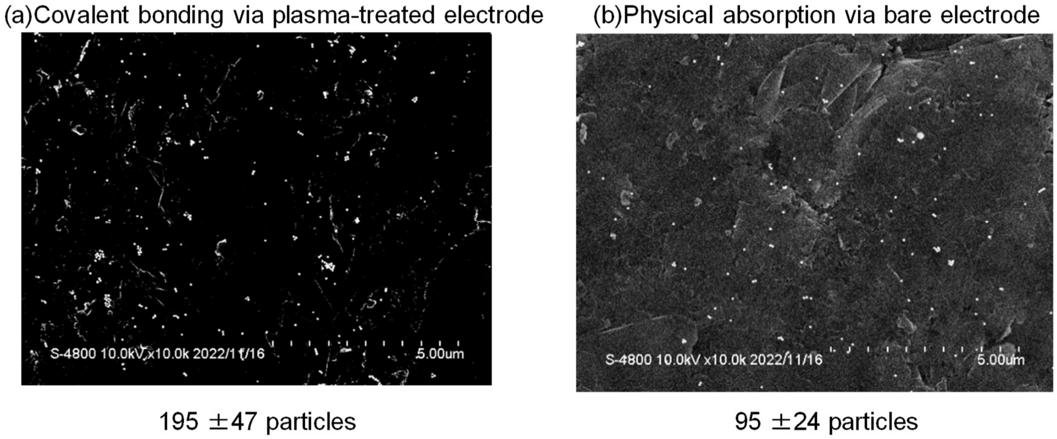

Figure S2: Scanning electron microscopy images observed from after antigen–antibody reaction on plasma-treated electrode (a) and bare electrode (b).

Table S1: Signal values used in the calibration curves.

O_2-plasma treated electrode

IgA [ng/mL]	n1	n2	n3	ave	stdev
100	1.77	1.80	1.64	1.74	0.09
50	1.87	1.80	1.42	1.69	0.24
10	0.53	0.44	0.43	0.47	0.05
5	0.14	0.27	0.13	0.18	0.08
0	0.00	0.01	0.01	0.01	0.01

Bare electrode

IgA [ng/mL]	n1	n2	n3	ave	stdev
100	1.46	1.37	1.93	1.59	0.30
50	1.47	1.40	1.30	1.39	0.09
10	0.56	0.85	0.80	0.74	0.16
5	0.55	0.45	0.60	0.53	0.07
0	0.32	0.34	0.33	0.33	0.01
